# The Individualized Diet and Exercise Adherence Pilot Trial (IDEA-P) in prostate cancer patients undergoing androgen deprivation therapy: study protocol for a randomized controlled trial

**DOI:** 10.1186/1745-6215-15-354

**Published:** 2014-09-09

**Authors:** Brian C Focht, Alexander R Lucas, Elizabeth Grainger, Christina Simpson, Jennifer M Thomas-Ahner, Steven K Clinton

**Affiliations:** Exercise and Behavioral Medicine Laboratory, Kinesiology, The Ohio State University, Columbus, OH USA; Comprehensive Cancer Center, The Ohio State University, 305 W 17th Avenue, Columbus, OH 43210 USA; Department of Internal Medicine, Division of Medical Oncology, The Ohio State University, Columbus, OH USA

**Keywords:** Androgen deprivation, Diet, Exercise, Functional limitations, Prostate cancer

## Abstract

**Background:**

Androgen deprivation therapy (ADT) is the foundation of treatment for men with metastatic prostate cancer and is now frequently incorporated into multimodality strategies for the curative treatment of locally advanced prostate cancer. Nevertheless, the catabolic effects of ADT result in meaningful adverse effects on physiological and quality of life outcomes, which may, in turn, increase the risk of functional decline, frailty, cardiovascular disease, and metabolic syndrome. Recent evidence demonstrates that lifestyle intervention promoting change in exercise and dietary behaviors is a promising approach, and may offset, or even reverse, the adverse effects accompanying ADT. Unfortunately, the limited existing studies of the effects of exercise and dietary interventions targeting patients with prostate cancer on ADT are characterized by high attrition rates and poor postintervention maintenance of treatment effects. Consequently, the Individualized Diet and Exercise Adherence Pilot Trial (IDEA-P) is designed to contrast the effects of a lifestyle intervention designed to promote independent self-management of exercise and dietary behavior with those of standard care disease management approach in the treatment of prostate cancer.

**Methods/Design:**

A total of 40 patients with prostate cancer undergoing ADT will be randomly assigned to lifestyle intervention or standard care. Outcomes of interest in IDEA-P include changes in self-reported and objectively assessed physical function and physical activity, dietary behavior, body composition, muscular strength, and quality of life. Outcomes will be obtained at baseline, 2-month, and 3-month assessments by trial personnel blinded to participants’ randomization assignment.

**Discussion:**

Findings from this study will establish the feasibility and preliminary efficacy of an innovative lifestyle intervention designed to promote progressively independent self-regulated exercise and dietary behavior change in the treatment of patients with prostate cancer undergoing ADT.

**Trial registration:**

ClinicalTrials.gov NCT02050906.

**Electronic supplementary material:**

The online version of this article (doi:10.1186/1745-6215-15-354) contains supplementary material, which is available to authorized users.

## Background

Despite the well-established therapeutic efficacy of androgen deprivation therapy (ADT) in the treatment of prostate cancer [1], it has become increasingly evident that men on ADT endure lingering adverse effects as a ‘trade-off’ for more effective cancer control and increased longevity. The catabolic effects of ADT result in significant adverse effects, including loss of lean muscle mass, increased fat mass, reduced muscle strength, and lower bone mineral density, which place men undergoing ADT at greater risk of functional decline and frailty [2–9]. Emerging evidence also suggests that ADT increases the risk of cardiovascular disease and metabolic syndrome. As prolonged administration of ADT becomes increasingly common, many men will be required to cope with lasting treatment-related side-effects that could meaningfully compromise their physical function and quality of life. Prostate cancer is estimated to be the cause of over half a million disability-adjusted life years [10–12]. Thus, defining the feasibility and efficacy of innovative interventions that preserve functional abilities and quality of life and attenuate risk for chronic disease are primary clinical considerations for patients with prostate cancer on ADT [2, 7, 13–17].

Exercise consistently results in improvements in relevant physiologic and patient-reported outcomes across a variety of cancer patients and survivors [10, 18–30]. Findings from recent randomized controlled exercise intervention trials in patients with prostate cancer undergoing ADT also suggest that exercise yields significant, clinically meaningful improvements in muscular strength, physical function, and quality of life [31]. Collectively, these findings provide strong support for the beneficial role of exercise as an adjuvant, supportive care intervention in the treatment of patients with prostate cancer. Despite the clear benefits accompanying exercise, it is also well established within the weight management literature that modifying both energy expenditure via increased physical activity and energy intake through changes in dietary behavior is integral to successful behavioral weight management interventions [32–34]. Primary adverse effects of ADT are increases in body fat or bodyweight and decreases in muscle mass or strength, which in turn, place patients with prostate cancer at increased risk of functional decline, cardiovascular disease, and metabolic syndrome. Thus, the synergistic benefits of concomitant change in both exercise and dietary behavior could represent an optimal lifestyle intervention approach for offsetting the adverse effects experienced by patients with prostate cancer during ADT.

Consistent with this position, recent findings revealed that lifestyle interventions combining primarily supervised exercise and dietary advice yielded significant improvements in aerobic fitness, muscular strength, self-reported exercise participation [35], fatigue, quality of life [36], and select bodyweight-related outcomes [37] relative to standard care [35, 36] or metformin treatment [37] in patients with prostate cancer undergoing prolonged ADT. Results of these trials are clearly important in that they provide the first evidence of the feasibility and preliminary efficacy of implementing lifestyle interventions combining exercise and dietary modifications in the treatment of patients with prostate care on ADT. Unfortunately, despite these promising findings, 2 of the studies were characterized by high attrition rates, of 44% [35] and 32% [36], respectively, at post-treatment follow-up. Additionally, in one study, clinically meaningful improvements in quality of life accompanying the 12-week lifestyle intervention were not maintained at 6-month follow-up [36].

The deterioration of benefits accompanying lifestyle interventions have been proposed to be directly related to poor post-treatment adherence to exercise and dietary behavior change [38–40]. Thus, given that adherence to the desired behavior changes is an essential determinant of the efficacy of lifestyle interventions, these findings underscore the pressing need to explore novel approaches to promoting successful adoption and maintenance of independent exercise and dietary behavior among patients with prostate cancer. It has been proposed that high attrition and poor adherence observed in lifestyle interventions may be attributable to a failure to provide patients with the self-regulatory skills necessary to adopt and maintain independent lifestyle behavior change [40]. One new approach based on social cognitive theory and the group dynamics literature [41], a group-mediated cognitive behavioral (GMCB) lifestyle intervention, has recently produced superior adherence to exercise and dietary behavior change and also yielded significant improvements in a variety of clinically relevant outcomes for patients with prostate cancer in randomized trials targeting chronic disease patients [40, 42–44]. The GMCB intervention couples exercise and dietary behavior change with self-regulatory skills counseling, to promote independent maintenance of lifestyle behavior change and sustain intervention-induced improvements in relevant outcomes. Although these findings suggest that this approach holds promise for improving the utility of lifestyle exercise and dietary interventions targeting patients with prostate cancer, the feasibility and efficacy of implementing this approach in the treatment of patients with prostate cancer undergoing ADT has not been investigated. Therefore, the purpose of this pilot trial is to examine the feasibility and preliminary efficacy of implementing the GMCB exercise and dietary lifestyle intervention in the treatment of patients with prostate cancer undergoing ADT.

## Methods/Design

### Overview

**Figure 1:**
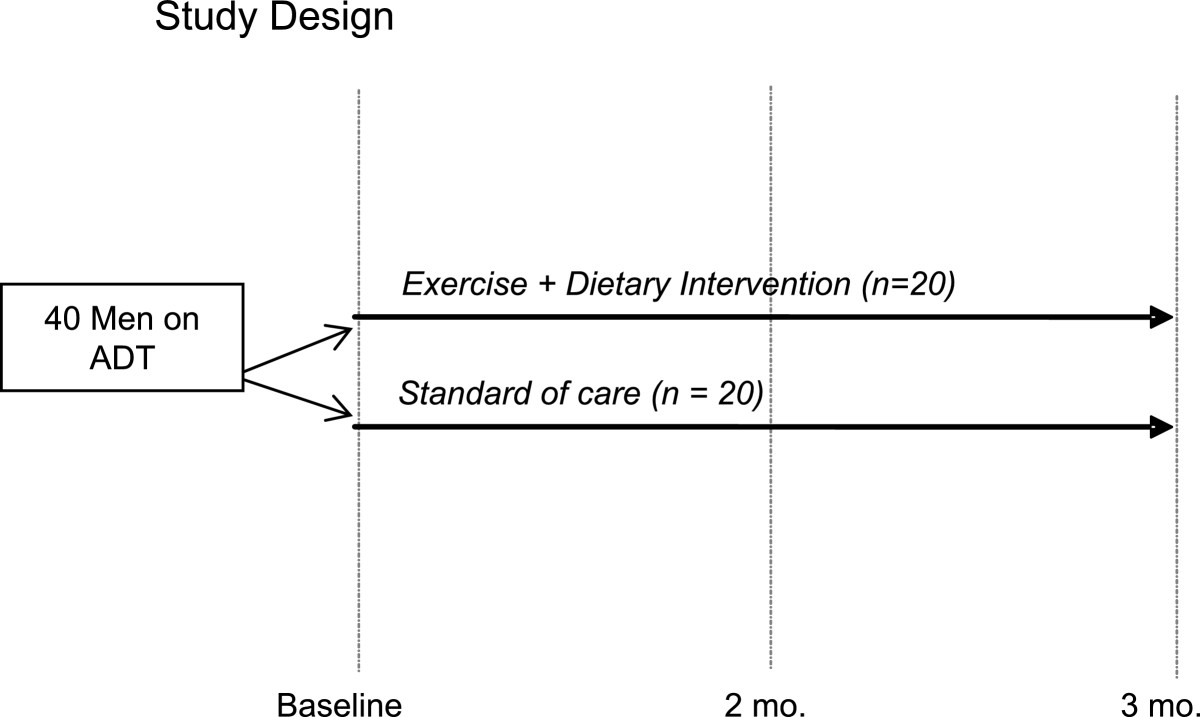
**Design of the IDEA-P trial.**

The Individualized Diet and Exercise Adherence Pilot Trial (IDEA-P) is a two-arm, single-blind randomized controlled pilot trial designed to examine the effects of a GMCB lifestyle intervention combining exercise and dietary intervention approaches relative to those of a standard care disease management approach on inpatients with prostate cancer undergoing ADT (see Figure 1). Primary objectives of IDEA-P are to determine the feasibility of delivering this specific lifestyle intervention approach to patients with prostate cancer undergoing ADT and to explore the preliminary efficacy of the lifestyle intervention for improving clinically relevant physiologic and quality of life outcomes and the short-term adoption and maintenance of independent, self-regulated exercise and dietary behavior change for men undergoing androgen suppression therapy. A total of 40 patients with prostate cancer on ADT will be randomly assigned to either lifestyle intervention (*n* = 20) or standard care (*n* = 20) arms. Given that this is a pilot study, it should be recognized that the target patient accrual does not provide optimal statistical power but is adequate to obtain effect size estimates necessary to inform the design of a subsequent optimally powered randomized controlled lifestyle intervention trial. Assessments of the primary and secondary outcomes will be obtained by study staff who are blinded to treatment arm assignment at baseline, 2-month, and 3-month follow-up screening visits.

### Participant eligibility

The inclusion and exclusion criteria are designed to target the recruitment of sedentary patients with prostate cancer undergoing ADT who are sufficiently healthy to participate in a supervised center-based exercise intervention involving both resistance and aerobic exercise. To be eligible to participate in the IDEA-P trial, volunteers will meet the following inclusion criteria: (a) histologically defined diagnosis of prostate cancer based upon pathology reports and staging studies; (b) currently undergoing ADT with a planned course of at least 3 months of continuous therapy; (c) sedentary activity pattern with less than 60 min of structured exercise participation per week during the past 6 months; (d) free of any serious medical condition that precludes safe participation in an exercise program, such as coronary artery disease, severe hypertension, peripheral vascular disease, stroke, congestive heart failure, chronic obstructive pulmonary disease, insulin-dependent diabetes, psychiatric disease, renal disease, liver disease, active cancer other than skin cancer, and anemia; (e) consent to participate from the treating oncologist and primary care physician; (f) willingness to accept randomization and undergo the testing and intervention procedures.

### Recruitment and randomization

Recruitment strategies include direct referral to study investigators from physicians at the Genitourinary Oncology Disease Unit of the James Cancer Hospital and Ohio State University Comprehensive Cancer Center and placement of study-related advertisements and informational brochures in cancer center oncologists’ offices and cancer support newsletters. Volunteers interested in participating in the study will complete a telephone screening to verify eligibility. Participants determined to be eligible following the completion of the phone screening interview are then scheduled for the baseline screening visit. Eligible participants are randomly assigned with equal probability to each of the two treatment arms, using a 1:1 ratio, following the completion of the baseline screening visit. The computer-generated randomization allocation sequence is sequentially numbered and sealed in opaque envelopes. The randomization allocation sequence is also concealed from the study staff responsible for conducting the baseline assessments.

### Informed consent

Approval of trial protocol and informed consent documents has been obtained from the Ohio State University Cancer Institutional Review Board (Project number 2012 C008) prior to the initiation of recruitment procedures. All participants complete informed consent forms and Health Insurance Portability and Accountability Act authorization forms prior to participation in the trial.

### Measures

Assessments of all study measures are obtained at baseline, 2-month, and 3-month follow-up screening visits using measures with well-established validity and reliability, demonstrated in prior exercise intervention studies [43, 44]. Given that IDEA-P is a single-blind pilot trial, the outcome assessments are obtained by trained study personnel who are blinded to participants’ treatment assignment.

### Outcome assessments

#### Functional battery

The functional battery includes measures of both self-reported physical function and objective indices of functional performance. Self-reported functional limitations will be measured using the abbreviated Late-Life Function and Disability Inventory [45]. Functional performance will be assessed using three valid and reliable timed performance-related mobility tasks: a 400 meter walk, a stair climb, and a lift-and-carry task [26, 27, 46, 47]. The 400 meter walk test is completed in a corridor with two cones spaced 20 meters apart. Individuals are instructed to walk as quickly as they can and the time to complete ten laps around the cones is recorded as the performance measure. The stair climb task involves ascending a set of eight stairs, turning around at the top, and then descending. Participants are instructed to complete the task as quickly as they can and performance is measured as the total time (in seconds) necessary to complete the task. The lift-and-carry test is a simulated common daily activity test involving picking up a 10 lb container from a shelf, walking 10 feet around a cone, and returning the container to the starting position on the shelf. Participants are instructed to complete the task as quickly as they can and performance is measured as the total time (in seconds) necessary to complete the task.

#### Mobility-related self-efficacy

Mobility-related self-efficacy is assessed by asking participants to rate their confidence in successfully completing incrementally more challenging amounts of the 400 meter walk and stair climb tasks. For walking self-efficacy, participants are asked to rate their confidence on a 0 (no confidence at all) to 10 (completely confident) scale in completing two, four, six, eight, and ten laps around the cones without stopping. For both stair climb and lift-and-carry task self-efficacy, participants rate their confidence in successfully completing two, four, six, eight, and ten trips on the stairs without stopping. Mobility-related self-efficacy scores are calculated for each task by summing the total, dividing by the total number of ratings, and multiplying by ten to yield a score ranging from 0 to 100. This hierarchical procedure for assessing mobility-related self-efficacy is consistent with Bandura’s recommendations [41] and has been shown to be valid and reliable in prior exercise intervention trials targeting older adults [48].

#### Muscular strength

Muscular strength will be assessed using standardized one-repetition-maximum (1RM) testing protocols for the chest press and leg extension exercises [49, 50]. These 1RM tests are the standard by which muscular strength is evaluated and have been found to be safe for older adults. Participants are familiarized with the chest press and leg extension machines and receive instruction on proper form. Participants will begin 1RM testing for each exercise by complete a warm-up set of four to six repetitions. Participants will rate the difficulty of the set using a 10-point difficulty scale ranging from 1 (not at all difficult) to 10 (extremely difficult). The participant perceptions of difficulty rating are used to choose the first weight at which a 1RM test will be attempted. The participant will be asked to lift the weight once and to continue to perform single repetition lifts, separated by at least a 2-minute rest interval, until a maximum weight is reached and recorded as the 1RM.

#### Body composition

Body composition will be assessed using both the Bod Pod system (Life Measurement Inc., Concord, CA) and dual energy X-ray absorptiometry (iDXA; GE Health Care Lunar, Madison, WI). The Bod Pod system uses whole-body densitometry to determine body composition (body fat and lean body mass). Whole-body densitometry is based on the determination of body mass and body volume, since body density is equivalent to body weight divided by body volume. The Bod Pod has well-established validity and reliability as an assessment of body composition [51]. The iDXA scans were used to determine total body composition as well as percentage body fat and fat-free mass for the trunk, arms, legs, android, and gynoid subregions. The iDXA method also has well-established validity and reliability and has been used to assess body composition in prior studies among patients with prostate cancer undergoing ADT [31].

#### Physical activity and dietary behavior

Assessments of physical activity are obtained using objective and self-report measures. The LIFECORDER EX accelerometer (Suzuken Kenz Inc. Limited, Japan) is used to obtain an objective assessment of exercise and physical activity participation. Participants wear the LIFECORDER EX on their right hip attached to either a waistband or belt during all waking hours, except when showering, bathing, or swimming, for 7 consecutive days following the completion of the baseline screening visit. Participants record the times they put on and take off the LIFECORDER EX on a self-monitoring log. The LIFECORDER EX provides assessment of minutes of light, moderate, and vigorous physical activity participation as well as calculating total daily steps taken. Consistent with the metabolic demands for the targeted age group [44], the accelerometer was set for intensity levels of 3 to 6 metabolic equivalents (METs), corresponding to moderate intensity physical activity, and >6 METs, corresponding to vigorous intensity physical activity. Self-reported physical activity is assessed using the CHAMPS questionnaire [52] and the Leisure Time Exercise Questionnaire (LTEQ) [53]. The CHAMPS questionnaire is a 41-item measure developed specifically for the assessment of physical activity in adults 50 years and older. The CHAMPS measure yields estimates of total minutes of physical activity and energy expended per week in all physical activities of moderate or higher intensity. The LTEQ measures the self-reported frequency of exercise participation performed in one’s leisure time during a typical week. Participants are asked to report the number of strenuous, moderate, and mild bouts of exercise they perform during an average week. The LTEQ has been shown to demonstrate adequate reliability and validity in previous research [54]. Dietary behavior is assessed using the Food Frequency Questionnaire, developed by the Nutrition Assessment Shared Resource of Fred Hutchinson Cancer Research Center, and 3-day food records. The Food Frequency Questionnaire provides a validated assessment of long-term dietary patterns. The three-day diet records provide information on more recent diet habits and enable us to determine compliance to the weekly dietary goals established at the group nutrition counseling sessions, as well as general dietary changes during the intervention.

#### Quality of life

Assessments of global and disease-specific indices of quality of life will be obtained using several valid and reliable scales. The Medical Outcomes Study 36-item short-form Health Survey [55] is a generic measure of health-related quality of life, and consists of two norm-based composite scales (mental health and physical health) and eight subscales (physical functioning, mental health, role-physical, role-emotional, bodily pain, general health, vitality, and social functioning). The Satisfaction with Life Scale [56] is a five-item measure of global life satisfaction. Disease and function specific quality of life measures will include the Functional Assessment of Cancer Treatment-Prostate (FACT-P) survey [57], and the Satisfaction with Function and Appearance (SFA) scale [46]. The 12 items of the FACT-P scale designed to assess concerns specific to patients with prostate cancer will be assessed. The SFA scale comprises six items that assess satisfaction with physical abilities and three items assessing satisfaction with physical appearance. Symptoms of pain and fatigue will also be assessed. Pain will be measured with the short-form McGill Pain Questionnaire, a valid, reliable 15-item adjective checklist that captures sensory and affective dimensions of pain [58]. Fatigue will be measured with the Brief Fatigue Inventory, a psychometrically sound nine-item measure that assesses disease and treatment-related fatigue severity of cancer patients [59].

#### Feasibility measures

Descriptive statistics for assessments of select indicators of trial feasibility including recruitment rates, intervention adherence, adverse events, and retention rates will be calculated prospectively throughout the trial.

#### Procedures

Volunteers expressing an interest in participating in IDEA-P complete a phone screening interview to determine their eligibility for the study. Prior to participation in the trial, participants make a baseline screening visit, during which assessments of the all outcome measures are obtained. At the beginning of the baseline screening visit, inclusion criteria are verified and medical history, informed consent, and Health Insurance Portability and Accountability Act waiver documents are completed. Participants then complete the functional performance tasks and undergo body composition assessment and 1RM strength testing, followed by the questionnaire assessments. After completion of the questionnaires, participants receive oral and written instructions on how to wear the accelerometer. Participants wear the accelerometer for the next seven consecutive days and monitors are returned to trial staff via the US postal service. Upon completion of the baseline screening visit, participants are randomly assigned to one of the two treatment arms (lifestyle intervention or standard care). Clearance to exercise will be obtained from the primary care physician and treating oncologist prior to participation in either the lifestyle intervention or standard care treatment arms. Assessments of all outcomes are obtained using exactly the same procedures at 2-month and 3-month follow-up screening visits conducted by study staff blinded to participants’ treatment group assignment.

### Interventions

#### Exercise and dietary lifestyle intervention

The lifestyle intervention involves an 8-week, multi-component approach designed to facilitate exercise and dietary behavior change and promote adherence, independent of study staff, to these behavioral modifications. The exercise component involves a combination of aerobic and resistance exercise performed twice per week. The aerobic exercise stimulus consists of 10 to 30 minutes of exercise performed at a rating of perceived exertion ranging from 11 (fairly light) to 14 (moderately hard) on the participant’s choice of a treadmill, stationary cycle, or elliptical trainer. The resistance exercise stimulus involves performing three sets of 8 repeat maximum to 12 repeat maximum at a rating of perceived exertion ranging from 12 (moderately hard) to 15 (hard) of nine different exercises (leg extension, leg curl, chest press, lat pull-down, overhead press, triceps extension, bicep curl, calf raises, and abdominal crunch). Participants will take 1 to 2 minutes rest between each set and all sets will be performed in a symptom-limited manner. The exercise prescription is tailored to each individual’s abilities and exercise tolerance and capacity. Consequently, resistance exercise load, volume, and volume-load and aerobic exercise duration and intensity will be guided by participant’s exercise tolerance and gradually increased across the intervention to reach optimal targeted prescription ranges. All exercise sessions will last 1 hour in duration.

The GMCB activity counseling component, based on social cognitive theory, is also integrated with exercise to promote adoption and adherence to independent, self-regulated exercise participation and participant retention. Counseling is delivered via six small group sessions (20 to 30 minutes in duration) conducted once per week immediately following a center-based exercise session during months 1 and 2. Participants also receive four brief (20 minutes) individualized activity counseling sessions conducted via phone calls in months 1 to 3. The specific content of this component includes the promotion of group identity and social norms for activity; self-monitoring; goal setting; barrier problem solving; fostering social support; and reducing sedentary time. The purpose of the activity-related behavioral counseling component of the lifestyle intervention arm is to instruct patients with prostate cancer undergoing ADT on the use of self-regulatory skills necessary to adopt and maintain exercise, and, through the use of the group as an agent of behavioral change, facilitate motivation to develop and implement these behavioral skills in order to successfully plan and undertake increasingly frequent independent exercise and participation in physical activity. A basic principle underlying these contacts and their sequencing is one of gradually weaning participants from dependency on staff and the group program toward independent self-regulation of exercise. This process is one of a phased increase in the ratio of personal responsibility in conjunction with a phased decrease in staff, group, and clinic dependency. Thus, in contrast with most approaches in traditional exercise interventions, this approach places an emphasis on the regulation of behavior and social problem-solving barriers to promote independent exercise participation.

To foster the practice and mastery of the newly acquired exercise and behavioral skills and prevent participants from becoming dependent on the expertise of exercise staff to remain physically active, supervised center-based exercise decreases from two sessions per week in weeks 1 to 6 to one supervised session per week in weeks 7 and 8 of the intervention. During weeks 7 and 8, participants have the goal of completing one center-based exercise session independent of study staff supervision during each week. During month 3, participants will have the goal of completing two center-based exercise sessions independent of study staff supervision. Participants will be provided free access to the center-based exercise facility during all its standard operating hours during weeks 7 to 12. While the facility is supervised by trained fitness staff members during this time, the participants will have no supervisory contact with the study staff during these independent exercise sessions. The advantages of the approach of integrating counseling and the weaning from staff supervision are that they help participants actively apply their developing exercise and behavioral skills to exercise independently while concomitantly providing them access to the study’s exercise facility to facilitate completion of the independent exercise sessions during weeks 7 to 12. This also allows us to evaluate uptake of independent exercise adherence in months 2 and 3.

The dietary component of the lifestyle intervention includes 10 (30-minute) nutritional counseling sessions with a registered dietitian. The first eight counseling sessions will be conducted once per week immediately following a center-based exercise session during months 1 and 2. The two remaining sessions are conducted via biweekly phone calls during month 3. The specific dietary objectives are consistent with the therapeutic lifestyle changes recommended in the Adult Treatment Panel III Report of the National Cholesterol Education Program [60] and the American Institute of Cancer Research [61]. The nutrition intervention encourages reductions in portion size and caloric and fat consumption, together with a gradual transition from an animal-based diet to a more plant-rich diet while still incorporating animal foods, including milk and meat, with an emphasis on monitoring food proportion and portion size. Specific goals of the dietary component include: (a) reduction in energy intake by 500 to 1000 kcal per day; (b) reduction in total fats to 25 to 30%, saturated fats to 7%, and protein to 15% of total calories; (c) increase in fruit and vegetable consumption to five servings per day; (d) intake of three or more servings of whole grains per day and a gradual increase to at least 25 g of dietary fiber per day. The nutrition counseling uses the GMCB and motivational interviewing approaches that have been demonstrated to be an effective approach to promote behavior change in chronic disease [43] and cancer patients [58, 59]. The nutrition counseling also builds upon many of the cognitive behavioral self-management strategies utilized in the exercise intervention, including self-monitoring, building self-efficacy, goal setting, and anticipating and overcoming barriers to dietary behavior change.

### Standard care

Men randomized to the standard care arm receive usual treatment for prostate cancer, standard disease management education, as well as educational literature describing the American Institute of Cancer Research dietary and physical activity guidelines. To equate contact between treatment arms to levels consistent with similar, contemporary lifestyle intervention trials [62, 63], biweekly 20-minute phone calls will be made by Genitourinary Oncology clinic staff to men in the standard care arm, and will focus on routine aspects of self-management for prostate cancer. As an incentive to promote retention across the trial, men randomized to standard care will also receive two supervised exercise training sessions and dietary counseling sessions following the completion of the 3-month assessment. Men will complete assessments of all outcomes scheduled at baseline and in months 2 and 3.

### Statistical analysis

The primary hypotheses of the IDEA-P trial are: (1) the lifestyle intervention will be a safe, well-tolerated intervention that yields acceptable recruitment, adherence, retention, and adverse event rates; (2) the lifestyle intervention will result in superior improvements in physical function, muscular strength, body composition, and quality of life relative to standard care; and (3) the lifestyle intervention will successfully promote adoption and short-term maintenance of independent, self-regulated exercise and dietary behavior change at 3-month follow-up. Differences in the longitudinally gathered outcome data collected at 2- and-3 month follow-up assessments will be individually standardized by baseline values and evaluated using a weighted repeated measures analysis of variance statistical model adjusting for the effects of age and sex. All analyses will be conducted according to the intention to treat principle with last value carried forward imputation methods used to account for missing data. As noted previously, the target patient accrual does not provide optimal statistical power but is adequate to obtain effect size estimates necessary to inform the design of a subsequent optimally powered randomized controlled lifestyle intervention trial.

## Discussion

The IDEA-P trial is a single-blind, two-arm randomized controlled pilot trial evaluating the feasibility and preliminary efficacy of a GMCB lifestyle intervention approach combining individualized exercise, dietary modification and behavioral self-regulatory skills counseling in the treatment of patients with prostate cancer undergoing ADT. Given the integral role of ADT in the treatment of prostate cancer, there is a critical need to determine the benefits of supportive care approaches in reducing the adverse effects accompanying ADT. The synergistic benefits of lifestyle interventions combining exercise and dietary behavior change may be a particularly beneficial adjuvant treatment approach for offsetting the adverse effects experienced by patients with prostate cancer during ADT. Although recent findings suggest that lifestyle interventions combining exercise and dietary advice approaches yield significant improvements in clinically relevant outcomes for patients with prostate cancer undergoing ADT [35–37], some of these studies have been characterized by high attrition rates [35, 36] and poor postintervention maintenance of treatment effects [36]. Consequently, these findings suggest that novel approaches for improving successful adherence to independent self-regulation exercise and dietary behavior change are warranted.

The proposed pilot trial is innovative from both research and clinical practice perspectives. From a research perspective, IDEA-P is innovative in that it expands knowledge from existing studies of the benefits of lifestyle exercise and dietary interventions in patients with prostate cancer on ADT [35–37]. Notably, it is the first to determine the feasibility and efficacy of implementing a group-based lifestyle intervention, which has been effective in promoting both successful maintenance of independent exercise and dietary behavior change and superior changes in relevant functional and quality of life outcomes in other chronic disease patients [40, 42, 43], among men with prostate cancer undergoing ADT. There are also other methodological aspects of IDEA-P that make it innovative and different from extant research on prostate cancer. For example, implementing theory-based activity and dietary behavioral counseling to promote behavior change expands on the primarily psycho-educational advice-based approaches used in prior studies. Furthermore, whereas prior investigations have relied exclusively on self-reported physical activity assessments, which consistently overestimate exercise and physical activity participation, this study is the first to include a more accurate objective measure of physical activity. Collectively, these features distinguish IDEA-P from prior or ongoing lifestyle interventions targeting men on ADT. From a clinical perspective, we believe that determining the feasibility, safety, and preliminary efficacy of implementing this lifestyle intervention approach in men undergoing ADT will have meaningful future implications for clinical practice for patients with prostate cancer. The results of this feasibility study will inform the design of larger randomized controlled lifestyle intervention trials. If findings from larger scale efficacy trials demonstrate meaningful benefits of implementing this lifestyle exercise and dietary intervention in the treatment of patients with prostate cancer undergoing ADT, such results could provide the evidence necessary to alter current standard care practices toward the inclusion of exercise and dietary interventions in the routine clinical treatment of patients with prostate cancer.

Although findings from the IDEA-P trial could yield meaningful implications for the role of lifestyle interventions as an adjuvant behavioral approach for the treatment of patients with prostate cancer, there are select study limitations that should be acknowledged. Given that this is a pilot trial intended to determine the safety, feasibility, and preliminary efficacy of delivering the group-based behavioral lifestyle intervention during ADT, the sample size does not provide sufficient power to detect meaningful differences in all relevant outcomes of interest. Additionally, there are clearly other clinically relevant outcomes, such as select biomarkers of prostate cancer and chronic disease, that may be favorably influenced by the lifestyle intervention but are not assessed in this pilot trial.

In summary, determining the feasibility and preliminary efficacy of the lifestyle intervention relative to the effects of standard care could have significant implications for the treatment of patients with prostate cancer on ADT. Findings from the present pilot trial will also provide effect size estimates necessary to inform the design of a subsequent, large-scale definitive lifestyle intervention efficacy trial, the results of which would fill a critical gap in knowledge, addressing the utility of implementing exercise and dietary modification in the treatment of patients with prostate cancer undergoing ADT.

## Trial status

The IDEA-P trial is active with patient recruitment and intervention delivery currently ongoing.

## Authors’ original submitted files for images

Below are the links to the authors’ original submitted files for images. Authors’ original file for figure 1

## Abbreviations

1RM: one-repetition-maximum
ADT: androgen deprivation therapy
FACT-P: Functional Assessment of Cancer Treatment - Prostate
GMCB: group-mediated cognitive behavioral
IDEA-P: individualized diet and exercise adherence trial - pilot
iDXA: dual energy X-ray absorptiometry
LTEQ: Leisure Time Exercise Questionnaire
MET: metabolic equivalent
SFA: Satisfaction with Function and Appearance Scale.

## References

[1] Cooperberg MR, Moul JW, Carroll PR (2005). The changing face of prostate cancer. J Clin Oncol.

[2] Basaria S, Lieb J, Tang AM, DeWeese T, Carducci M, Eisenberger M, Dobs AS (2002). Long-term effects of androgen deprivation therapy in prostate cancer patients. Clin Endocrinol (Oxf).

[3] Boxer RS, Kenny AM, Dowsett R, Taxel P (2005). The effect of 6 months of androgen deprivation therapy on muscle and fat mass in older men with localized prostate cancer. Aging Male.

[4] Bylow K, Dale W, Mustian K, Stadler WM, Rodin M, Hall W, Lachs M, Mohile SG (2008). Falls and physical performance deficits in older patients with prostate cancer undergoing androgen deprivation therapy. Urology.

[5] Bylow K, Mohile SG, Stadler WM, Dale W (2007). Does androgen-deprivation therapy accelerate the development of frailty in older men with prostate cancer?: a conceptual review. Cancer.

[6] Chen Z, Maricic M, Nguyen P, Ahmann FR, Bruhn R, Dalkin BL (2002). Low bone density and high percentage of body fat among men who were treated with androgen deprivation therapy for prostate carcinoma. Cancer.

[7] Dacal K, Sereika SM, Greenspan SL (2006). Quality of life in prostate cancer patients taking androgen deprivation therapy. J Am Geriatr Soc.

[8] Diamond TH, Higano CS, Smith MR, Guise TA, Singer FR (2004). Osteoporosis in men with prostate carcinoma receiving androgen-deprivation therapy: recommendations for diagnosis and therapies. Cancer.

[9] Levine GN, D’Amico AV, Berger P, Clark PE, Eckel RH, Keating NL, Milani RV, Sagalowsky AI, Smith MR, Zakai N (2010). Androgen-deprivation therapy in prostate cancer and cardiovascular risk. A science advisory from the American Heart Association, American Cancer Society, and American Urological Association: endorsed by the American Society for Radiation Oncology. Circulation.

[10] Courneya KS, Stevinson C, Vallance JKH (2007). Exercise and Psychosocial Issues for Cancer Survivors.

[11] Crawford ED (2003). Epidemiology of prostate cancer. Urology.

[12] Ruchlin HS, Pellissier JM (2001). An economic overview of prostate carcinoma. Cancer.

[13] American Cancer Society (2005). Cancer Facts and Figures 2005.

[14] American Cancer Society (2006). Cancer Facts and Figures 2006.

[15] Holzbeierlein JM, McLaughlin MD, Thrasher JB (2004). Complications of androgen deprivation therapy for prostate cancer. Curr Opin Urol.

[16] Joly F, Alibhai SM, Galica J, Park A, Yi QL, Wagner L, Tannock IF (2006). Impact of androgen deprivation therapy on physical and cognitive function, as well as quality of life of patients with nonmetastatic prostate cancer. J Urol.

[17] Kornblith AB, Herr HW, Ofman US, Scher HI, Holland JC (1994). Quality of life of patients with prostate cancer and their spouses. The value of a data base in clinical care. Cancer.

[18] Carmack Taylor CL, Smith MA, de Moor C, Dunn AL, Pettaway C, Sellin R, Charnsangavej C, Hansen MC, Gritz ER (2004). Quality of life intervention for prostate cancer patients: design and baseline characteristics of the active for life after cancer trial. Control Clin Trials.

[19] Courneya KS (2003). Exercise in cancer survivors: an overview of research. Med Sci Sports Exerc.

[20] Courneya KS, Friedenreich CM (1997). Relationship between exercise pattern across the cancer experience and current quality of life in colorectal cancer survivors. J Altern Complement Med.

[21] Dimeo FC, Tilmann MH, Bertz H, Kanz L, Mertelsmann R, Keul J (1997). Aerobic exercise in the rehabilitation of cancer patients after high dose chemotherapy and autologous peripheral stem cell transplantation. Cancer.

[22] Galvao DA, Newton RU (2005). Review of exercise intervention studies in cancer patients. J Clin Oncol.

[23] Galvao DA, Taaffe DR, Spry N, Joseph D, Turner D, Newton RU (2009). Reduced muscle strength and functional performance in men with prostate cancer undergoing androgen suppression: a comprehensive cross-sectional investigation. Prostate Cancer Prostatic Dis.

[24] Nelson ME, Fiatarone MA, Morganti CM, Trice I, Greenberg RA, Evans WJ (1994). Effects of high-intensity strength training on multiple risk factors for osteoporotic fractures. A randomized controlled trial. JAMA.

[25] Nieman DC, Cook VD, Henson DA, Suttles J, Rejeski WJ, Ribisl PM, Fagoaga OR, Nehlsen-Cannarella SL (1995). Moderate exercise training and natural killer cell cytotoxic activity in breast cancer patients. Int J Sports Med.

[26] Peters C, Lotzerich H, Niemeier B, Schule K, Uhlenbruck G (1994). Influence of a moderate exercise training on natural killer cytotoxicity and personality traits in cancer patients. Anticancer Res.

[27] Peters C, Lotzerich H, Niemeir B, Schule K, Uhlenbruck G (1995). Exercise, cancer and the immune response of monocytes. Anticancer Res.

[28] Schmitz KH, Holtzman J, Courneya KS, Masse LC, Duval S, Kane R (2005). Controlled physical activity trials in cancer survivors: a systematic review and meta-analysis. Cancer Epidemiol Biomarkers Prev.

[29] Segal RJ, Reid RD, Courneya KS, Malone SC, Parliament MB, Scott CG, Venner PM, Quinney HA, Jones LW, D’Angelo ME, Wells GA (2003). Resistance exercise in men receiving androgen deprivation therapy for prostate cancer. J Clin Oncol.

[30] Durak EP, Lilly PC (1998). The application of an exercise and wellness program for cancer patients: a preliminary outcomes report. J Strength Cond Res.

[31] Thorsen L, Courneya KS, Stevinson C, Fossa SD (2008). A systematic review of physical activity in prostate cancer survivors: outcomes, prevalence, and determinants. Support Care Cancer.

[32] Goldberg JH, King AC (2007). Physical activity and weight management across the lifespan. Annu Rev Public Health.

[33] Jakicic JM, Otto AD (2006). Treatment and prevention of obesity: what is the role of exercise?. Nutr Rev.

[34] Wing RR (2000). Cross-cutting themes in maintenance of behavior change. Health Psychol.

[35] Bourke L, Doll H, Crank H, Daley A, Rosario D, Saxton JM (2011). Lifestyle intervention in men with advanced prostate cancer receiving androgen suppression therapy: a feasibility study. Cancer Epidemiol Biomarkers Prev.

[36] Bourke L, Gilbert S, Hooper R, Steed LA, Joshi M, Catto JW, Saxton JM, Rosario DJ (2014). Lifestyle changes for improving disease-specific quality of life in sedentary men on long-term androgen-deprivation therapy for advanced prostate cancer: a randomised controlled trial. Eur Urol.

[37] Nobes JP, Langley SE, Klopper T, Russell-Jones D, Laing RW (2012). A prospective, randomized pilot study evaluating the effects of metformin and lifestyle intervention on patients with prostate cancer receiving androgen deprivation therapy. BJU Int.

[38] Ettinger WH, Burns R, Messier SP, Applegate W, Rejeski WJ, Morgan T, Shumaker S, Berry MJ, O’Toole M, Monu J, Craven T (1997). A randomized trial comparing aerobic exercise and resistance exercise with a health education program in older adults with knee osteoarthritis. The Fitness Arthritis and Seniors Trial (FAST). JAMA.

[39] Focht BC (2006). Effectiveness of exercise interventions in reducing pain symptoms among older adults with knee osteoarthritis: a review. J Aging Phys Act.

[40] Rejeski WJ, Brawley LR, Ambrosius WT, Brubaker PH, Focht BC, Foy CG, Fox LD (2003). Older adults with chronic disease: benefits of group-mediated counseling in the promotion of physically active lifestyles. Health Psychol.

[41] Bandura A (1997). Self-Efficacy: The Exercise of Control.

[42] Focht BC, Garver MJ, Devor ST, Dials J, Rose M, Lucas AR, Emery CF, Hackshaw K, Rejeski WJ (2012). Improving maintenance of physical activity in older, knee osteoarthritis patients trial-pilot (IMPACT-P): Design and methods. Contemp Clin Trials.

[43] Rejeski WJ, Brubaker PH, Goff DC, Bearon LB, McClelland JW, Perri MG, Ambrosius WT (2011). Translating weight loss and physical activity programs into the community to preserve mobility in older, obese adults in poor cardiovascular health. Arch Intern Med.

[44] Rejeski WJ, Fielding RA, Blair SN, Guralnik JM, Gill TM, Hadley EC, King AC, Kritchevsky SB, Miller ME, Newman AB, Pahor M (2005). The lifestyle interventions and independence for elders (LIFE) pilot study: design and methods. Contemp Clin Trials.

[45] McAuley E, Konopack JF, Motl RW, Rosengren K, Morris KS (2005). Measuring disability and function in older women: psychometric properties of the Late-Life Function and Disability Instrument. J Gerontol A Biol Sci Med Sci.

[46] Reboussin BA, Rejeski WJ, Martin K, Callahan K, Dunn AL, King AC, Sallis JF (2000). Correlates of satisfaction with body function and body appearance in older aged adults: The activity counseling trial. Psychol Health.

[47] Rejeski WJ, Ettinger WH, Schumaker S, James P, Burns R, Elam JT (1995). Assessing performance-related disability in patients with knee osteoarthritis. Osteoarthritis Cartilage.

[48] Focht BC, Rejeski WJ, Ambrosius WT, Katula JA, Messier SP (2005). Exercise, self-efficacy, and mobility performance in overweight and obese older adults with knee osteoarthritis. Arthritis Rheum.

[49] Baechle TR, Earle RW (2008). Essentials of Strength Training and Conditioning.

[50] American College of Sports Medicine (2010). ACSM’s Guidelines for Exercise Testing and Prescription.

[51] Heymsfield SB, Lichtman S, Baumgartner RN, Wang J, Kamen Y, Aliprantis A, Pierson RN (1990). Body composition of humans: comparison of two improved four-compartment models that differ in expense, technical complexity, and radiation exposure. Am J Clin Nutr.

[52] Stewart AL, Mills KM, King AC, Haskell WL, Gillis D, Ritter PL (2001). CHAMPS physical activity questionnaire for older adults: outcomes for interventions. Med Sci Sports Exerc.

[53] Godin G, Shephard RJ (1985). A simple method to assess exercise behavior in the community. Can J Appl Sport Sci.

[54] Jacobs DR, Ainsworth BE, Hartman TJ, Leon AS (1993). A simultaneous evaluation of 10 commonly used physical activity questionnaires. Med Sci Sports Exerc.

[55] Ware JE, New England Medical Center Hospital (1994). Health I: SF-36 Physical and Mental Health Summary Scales: A User’s Manual.

[56] Diener E, Emmons RA, Larsen RJ, Griffin S (1985). The Satisfaction with Life Scale. J Pers Assess.

[57] Esper P, Mo F, Chodak G, Sinner M, Cella D, Pienta KJ (1997). Measuring quality of life in men with prostate cancer using the functional assessment of cancer therapy-prostate instrument. Urology.

[58] Melzack R (1987). The short-form McGill Pain Questionnaire. Pain.

[59] Mendoza TR, Wang XS, Cleeland CS, Morrissey M, Johnson BA, Wendt JK, Huber SL (1999). The rapid assessment of fatigue severity in cancer patients: use of the Brief Fatigue Inventory. Cancer.

[60] Expert Panel on Detection (2001). Executive summary of the third report of the National Cholesterol Education Program (NCEP) expert panel on detection, evaluation, and treatment of high blood cholesterol in adults (adult treatment panel III). JAMA.

[61] World Cancer Research Fund (2007). Food, Nutrition and the Prevention of Cancer: A Global Perspective.

[62] Felson DT, Naimark A, Anderson J, Kazis L, Castelli W, Meenan RF (1987). The prevalence of knee osteoarthritis in the elderly. The Framingham Osteoarthritis Study. Arthritis Rheum.

[63] Davis MA, Ettinger WH, Neuhaus JM (1990). Obesity and osteoarthritis of the knee: evidence from the National Health and Nutrition Examination Survey (NHANES I). Semin Arthritis Rheum.

