# Supplementary figures and images for: The Individualized Diet and Exercise Adherence Pilot Trial (IDEA-P) in prostate cancer patients undergoing androgen deprivation therapy: study protocol for a randomized controlled trial
Source: Trials. 2014 Sep 9;15:354. doi: 10.1186/1745-6215-15-354 (PMC4175627; doi:10.1186/1745-6215-15-354)

# Study Design

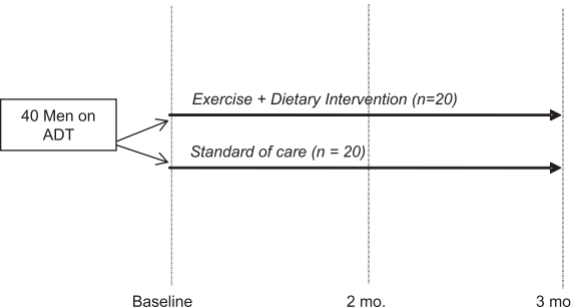

Supplement: Supplementary file 1 — Authors’ original file for figure 1 [file 13063_2014_2229_MOESM1_ESM.pdf]
